# Stem Cells from Human Exfoliated Deciduous Teeth Ameliorate Diabetic Nephropathy In Vivo and In Vitro by Inhibiting Advanced Glycation End Product-Activated Epithelial-Mesenchymal Transition

**DOI:** 10.1155/2019/2751475

**Published:** 2019-12-01

**Authors:** Nanquan Rao, Xiaotong Wang, Jing Xie, Jingzhi Li, Yue Zhai, Xiaoxia Li, Tengjiaozi Fang, Yuanyuan Wang, Yuming Zhao, Lihong Ge

**Affiliations:** ^1^Department of Pediatric Dentistry, Peking University School and Hospital of Stomatology, 22 Zhongguancun Avenue South, Haidian District, Beijing 100081, China; ^2^Department of Oral Emergency Department, Peking University School and Hospital of Stomatology, 22 Zhongguancun Avenue South, Haidian District, Beijing 100081, China; ^3^Department of Stomatology, Shenzhen Children's Hospital, No. 7019, Yitian Road, Shenzhen 518026, China

## Abstract

Diabetic nephropathy (DN) is a major cause of chronic kidney disease. It has been proven that mesenchymal stem cells (MSCs) have therapeutic effects on kidney disease. Stem cells from human exfoliated deciduous teeth (SHED) are MSCs that are derived from dental pulps in exfoliated deciduous teeth from young patients and therefore have a high proliferation rate and an easy access. Hence, we aimed to explore the effect of SHED on DN in Goto-Kakizaki (GK) rats. SHED were administered via the tail vein. Blood glucose, serum triglycerides and cholesterol, body weight, and urinary albumin were measured before and after administration. At 8 weeks after administration, real-time PCR, immunohistochemistry (IHC), and electron microscopy were employed to examine pathological changes in glomerular and tubulointerstitial tissue. Kidney weight and serum IL-1, IL-10, TNF-*α*, TGF-*β*, and HGF levels were measured. SHED engraftment in the kidneys was detected by transfecting green fluorescence protein (GFP). Type II epithelial-mesenchymal transition (EMT) in the tubule-interstitial and arteriolar regions has been reported to be an important pathological characteristic of DN. This study is the first to apply a transwell system for coculture to explore the effects of MSCs on the EMT of human proximal tubular epithelial (HK-2) cells. The effects of SHED on advanced glycation end product- (AGE-) activated EMT in HK-2 cells were explored by real-time PCR and western blot. At 8 weeks after administration, renal injury, including hyperglycemia, hyperlipidemia, increased urinary albumin excretion, ECM accumulation, and a fractional mesangial area, was dramatically attenuated. The serum levels of IL-1, TNF-*α*, and TGF-*β* were significantly downregulated, whereas the serum levels of IL-10 and HGF were upregulated by SHED. GFP expression confirmed the engraftment of SHED in diabetic kidneys. In addition, cocultured SHED inhibited AGE-induced EMT in HK-2 cells. In conclusion, SHED offer a novel potential effective therapeutic approach for ameliorating DN.

## 1. Introduction

Diabetes mellitus (DM) is estimated to become the 7th leading cause of death worldwide by 2030 [1]. DM is associated with complications that affect a patient's quality of life, such as cardiovascular diseases [2], retinopathy [3], diabetic neuropathy [4], and diabetic nephropathy (DN) [5]. DN affects more than 40% of patients with type 1 diabetes mellitus (T1DM) and type 2 diabetes mellitus (T2DM) [6]. DN remains the major cause of chronic kidney disease and approximately 50% of all cases of end-stage renal disease (ESRD) worldwide [7]. T2DM patients have a kidney transplantation rate of 9% [8]. DN is defined by increased urinary albumin excretion (UAE) in the absence of other renal diseases and is categorized into the following stages: microalbuminuria (UAE 20 *μ*g/min–199 *μ*g/min or 30–299 mg/24 h) and macroalbuminuria (UAE ≥ 200 *μ*g/minor ≥ 300 mg/24 h) [9]. The key pathological features of DN include gradual thickening of the glomerular basement membrane (GBM) and glomerular hypertrophy accompanied by mesangial matrix expansion with the accumulation of several matrix proteins, such as collagen I, laminin *β*1, and fibronectin, leading to a progressive reduction in the filtration surface of the glomerulus [10].

Type II EMT in the tubule-interstitial and arteriolar regions has been reported to be an important pathological characteristic of DN. When EMT occurs, HK-2 cells lose their epithelial phenotypes and acquire mesenchymal, fibroblast-like properties [11, 12]. AGEs, which mainly are the posttranscriptional modified proteins or lipids, bind to their multiligands, known as a receptor of advanced glycation end products (RAGE), which activates different kinases and NADPH oxidases leading increased levels of ROS and further promotes the synthesis of more AGEs, thereby triggering cell-damaging mechanisms[13–15].

DN progression can be prevented by tight glucose control, blood pressure control, renin-angiotensin-aldosterone system (RAAS) blockade, smoking cessation, and weight control. Although pancreatic transplantation can reverse the thickness of the glomerular and tubular basement membranes after five years of normoglycemia [16], this procedure is associated with adverse effects of immunosuppressive regimens [17] and an immunological risk that affect long-term survival [18].

Notably, MSC-based therapy has been considered a promising strategy for curing DM and related complications. MSCs are a population of self-renewable cells with the potential to differentiate into multiple cell types [19]. It is reported that human dental pulp stem cell (DPSC) transplantation through the IM route with a repeat dose is a superior option as a long-term treatment for diabetic neuropathy [20]. SHED are MSCs that are derived from dental pulps in exfoliated deciduous teeth from young patients and therefore have a higher proliferation rate [21]. SHED are more accessible for clinical application for noninvasive retrieval and are discarded as medical waste; thus, fewer ethical concerns exist. These cells also have proven potential to give rise to nondental cell lineages for use in the treatment of a broad spectrum of diseases, such as bone defects, traumatic brain injury, and T1DM [22–24]. Therefore, we hypothesize that SHED can be beneficial toward mitigating the progression of DN. In this study, GK rats, a nonobese and spontaneous (genetic) T2DM model, were first used to examine the effect of MSCs on DN. Additionally, the effects of MSCs on AGE-induced EMT in HK-2 cells were explored for the first time with a transwell system.

## 2. Materials and Methods

### 2.1. SHED and BMSCs Isolation, In Vitro Expansion, and Characterization

The SHED donors, aged 6 to 8 years, were from a pediatric clinic, while bone marrow-derived mesenchymal stem cells (BMSCs) donors were patients, aged 16 to 20 years, who had third-molar extractions.

According to the methods proposed by Miura et al. and practiced in our previous research [21, 25], the deciduous teeth were repeatedly washed with phosphate-buffered saline 3 times (PBS, Solarbio, Beijing, China); the pulp tissue was isolated, crushed, and digested with 3 mg/ml type I collagenase (Sigma-Aldrich, St Louis, Mo, USA) and 4 mg/ml dispase (Sigma-Aldrich, St Louis, Mo, USA) at a ratio of 1 : 1; incubated at 37°C for 1 h; and centrifuged at 1000 rpm/min for 5 min. The supernatant was discarded, and the cell pellet was resuspended in an appropriate volume of *α*-MEM (HyClone, GE Healthcare Life Sciences, South Logan, UT, USA) supplemented with 20% FBS (HyClone, GE Healthcare Life Sciences, South Logan, UT, USA) and 1% antibiotics (100 units/ml penicillin G and 100 g/ml streptomycin, Solarbio, Beijing, China) and filtered through a 70 *μ*m cell strainer. The cell suspension was seeded to 25 cm^2^ culture dishes containing *α*-MEM supplemented with 20% FBS and 1% antibiotics, and cells were cultured at 37°C in 5% CO_2_ for 3 days. The culture medium was changed every 3 or 4 days.

For the extraction of impacted wisdom teeth, the alveolar bone around a tooth was removed, and the bone marrow was exposed [26]. Subsequently, the aspirate was obtained from the marrow site using an 18G injection needle connected to a disposable syringe. Then, the aspirates were mixed immediately with *α*-MEM supplemented with 200 units/ml heparin (Solarbio, Beijing, China). The cells were centrifuged at 500 g/min for 5 min, resuspended with *α*-MEM, seeded at a density of 0.1 ml of aspirate/35 mm tissue culture dish, and maintained in 2 ml of *α*-MEM supplemented with 20% FBS and antibiotics at 37°C in 5% CO_2_ for 3 days. Thereafter, floating cells were removed, and attached cells were fed fresh medium, which was changed every 3 or 4 days.

When monolayer SHED and BMSCs confluence was observed, the cells were passaged at a ratio of 1 : 3 and cultured with *α*-MEM supplemented with 10% FBS and 1% antibiotics (mesenchymal cell-conditioned medium (MCM)) at 37°C in 5% CO_2_.

The surface marker profiles of the SHED and BMSCs at the third passage were verified by flow cytometry. Cells at the third passage were resuspended at a concentration of 1 × 10^6^ cells/ml in cold PBS containing 2% FBS prior to the addition of the following monoclonal antibodies: CD34-PE, CD45-PE, CD73-PE, CD90-FITC, CD105-FITC, and CD146-PE (Beckman Coulter, Brea, CA, USA). Unstained cells were used as the negative control. Finally, stained cells were analyzed using a Beckman Coulter flow cytometry system (FC500, Beckman Coulter, Brea, CA, USA).

### 2.2. Adipogenic and Osteogenic Differentiation

To induce adipogenic differentiation, we switched confluent cells to MCM supplemented with 200 *μ*M insulin (Sigma-Aldrich, St Louis, MO, USA), 100 *μ*M indomethacin (Sigma-Aldrich, St Louis, MO, USA), 1 *μ*M dexamethasone (Sigma-Aldrich, St Louis, MO, USA), and 100 *μ*g/ml 3-isobutyl-1-methyxathine (Sigma-Aldrich, St Louis, MO, USA). We replaced the medium every 2 to 3 days. The maturation of cells and lipid vesicle formation was assessed by performing Oil Red O (Sigma-Aldrich, St Louis, MO, USA) staining for 21 days in osteogenic medium.

To induce osteogenic differentiation, we switched confluent cells in MCM supplemented with 10 mM *β*-glycerophosphate (Sigma-Aldrich, St Louis, MO, USA), 50 *μ*g/ml ascorbate 2-phosphate (Sigma-Aldrich, St Louis, MO, USA), and 0.1 *μ*M dexamethasone. We replaced the medium every 2 to 3 days. Cells differentiated into hydroxyapatite-producing osteoblasts and mineralized matrix nodules were confirmed by Alizarin Red (Sigma-Aldrich, St Louis, MO, USA) staining for 21 days in osteogenic medium.

### 2.3. Cell Labeling for In Vivo Tracking

GFP was used as a cell tracker in the tracking experiments to assess the intrarenal locations of SHED and BMSCs. SHED and BMSCs were transduced with GFP by lentiviral vector according to the manufacturer's protocol (GenePharma, Shanghai, China). Briefly, the third passage of cells was cultured in 6-well plates at a concentration of 3-4 × 10^5^/well. When the cells reached 30-50% confluence, the regular medium was replaced with the enhanced infection solution and supplemented with the GFP lentiviral vector at a multiplicity of infection (MOI) of 100. After 12 h, the transfection medium was changed to regular medium, and the cells were expanded and collected for administration.

### 2.4. Animal Model and Groups

SPF-grade 12-week-old male GK rats weighing approximately 300-350 g were purchased from Changzhou Cavens Laboratory Animal Co., Ltd., Changzhou, Jiangsu, China. SPF-grade 12-week-old male Wistar rats weighing approximately 350-400 g were purchased from Beijing Vital River Laboratory Animal Technology Co., Ltd. (Beijing, China) for use as the normal control. Rats were maintained on a 12 h light : 12 h dark cycle with free access to rodent chow and water. After consumption of a high-fat diet for 2-4 weeks by GK rats, rats with nonfasting blood glucose levels ≥ 11.1 mM for 3 consecutive days were classified as DN rats.

**Figure 1:**
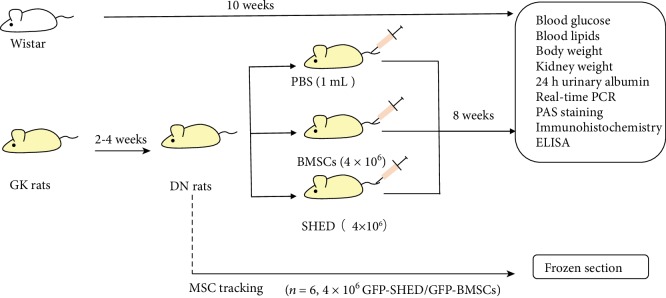
The experimental protocol. Two to four weeks after diabetes induction with a high-fat diet, rats presented with mild microalbuminuria (DN rats) and were divided into the PBS group (PBS infusion), SHED group (4 × 10^6^ cells/rat), and BMSC group (4 × 10^6^ cells/rat infusion). Six DN rats were chosen for cell tracking (4 × 10^6^ GFP-SHED/rat infusion or GFP-BMSCs/rat infusion) with frozen sections. Blood glucose, blood lipids, and body weight were measured before and after administration. At 8 weeks after administration, real-time polymerase chain reaction (PCR), immunohistochemistry, and electron microscopy were employed to examine pathological changes, and kidney weight and the serum levels of interleukin- (IL-) 1, IL-10, tumor necrosis factor- (TNF-) *α*, transforming growth factor- (TGF-) *β*, and hepatocyte growth factor (HGF) were also measured.

DN rats were randomly divided into 3 groups, and 6 normal nondiabetic rats were classified as the normal control group. DN rats were fed conventional chow. The 4 groups of rats received the following treatments: (1) normal nondiabetic rats received no treatment (normal group); (2) DN rats received SHED infusions (SHED group); (3) DN rats received 1 ml of PBS infusion (PBS group); and (4) DN rats received BMSCs infusions (BMSCs group). All rats in the 4 groups were observed for 8 weeks after treatment administration. In addition, 6 DN rats were transplanted with GFP-SHED or GFP-BMSCs for tracking purposes (see Figure 1).

### 2.5. MSC Administration

A total of 4 × 10^6^ cells, including BMSCs, SHED, GFP-BMSCs, and GFP-SHED, were resuspended in 1 ml of PBS and administered via the tail vein to each rat. The rats in PBS group received 1 ml of PBS each.

### 2.6. Physical and Biochemical Assessments

Body weight, fasting blood glucose, and nonfasting glucose were measured each week. Blood glucose levels were measured with the glucometer system Accu-Check Performa (Roche Diagnostic, Basel, Switzerland). Biochemical parameters were measured after diabetes induction (before MSCs administration) and at 8 weeks after MSC administration. The serum levels of triglycerides and cholesterol were determined with the Architect c8000 auto analyzer (Abbot, Lake Bluff, IL, USA). The kidney was decapsulated, and kidney weight was measured immediately after the rat was sacrificed. The renal mass index, determined by the ratio of the kidney weight to the body weight, was also calculated.

### 2.7. Renal Function Measurements

Rats were kept in metabolic cages for the collection of 24 h urine samples after diabetes induction (before MSCs administration) and at 8 weeks after MSCs administration. Urinary albumin levels were determined with the Architect c8000 auto analyzer.

### 2.8. Sample Collection

At 8 weeks after administration, rats were injected i.p. with 10% chloral hydrate (Solarbio, Beijing, China). Blood samples were collected from the retro-orbital vein, and rats were sacrificed and perfused through the left ventricle with 100 ml of cold PBS and then through the right ventricle with 20 ml of cold PBS before tissues were isolated by dissection. The kidneys were quickly removed, decapsulated, weighed, and dissected into two parts; one part was immediately frozen in liquid nitrogen and stored at −80°C for molecular biological studies, and the other part was stored in 4% paraformaldehyde for histopathological analysis. Additionally, renal cortices were cut into 1 mm^3^ pieces and fixed in 1.2% glutaraldehyde for analysis by electron microscopy.

At 2 weeks, 4 weeks, and 8 weeks after the administration of GFP-SHED or GFP-BMSCs, the rats were anesthetized, sacrificed, and perfused as described above. The kidneys were decapsulated and embedded in OCT (SAKURA, Tokyo, Japan) at -80°C.

### 2.9. Renal Gene Expression Analysis

Total RNA was extracted from kidney homogenates with the Trizol reagent (Invitrogen, Carlsbad, CA, USA) and reverse transcribed into cDNA with the PrimeScript RT reagent Kit (Takara Biotechnology, Dalian, Liaoning, China). RNA expression was assessed by real-time reverse transcriptase polymerase chain reaction (PCR) using the SYBR Green System (7300 Real time System, Applied Biosystems, Carlsbad, CA, USA) according to the manufacturer's protocol. cDNA was amplified by PCR using the following primers (forward primer, reverse primer): GADPH—5′-GACAACTTTGGCATCGTGGA-3′, 5′-ATGCAGGGATGATGTTCTTG-3′; *α*-SMA—5′-GCTGACGGATGCAGAAGGAG-3′, 5′-TGCTGGAAGGTGGAGAGAGAA-3′; Col1—5′-GACATGTTGAGCTTTGATTACCTC-3′, 5′-GGGACCCTTAGGCCATTGTGTA-3′; FN—5′-CTGAACCCAGTCCCGATGGTA-3′, 5′-CACGTCCAACGGCATGAAG-3′; laminin *β*—5′-CGGAAAGGAAGACACGAAGAA-3′, 5′-AGGACACCAAAGGCGAACA-3′; nephrin—5′-CTCCGTCTCCAGACCTTGGAAATA-3′, 5′-TGGATGGCTTTGGACACATGA-3′; and synaptopodin—5′-CCAGGGCAGAGCAGAGAGTAAA-3′, 5′-AGCCATCAATAAGCCCAGAAAA-3′.

### 2.10. Renal Histological and Immunohistochemical Analysis

Kidney tissue was embedded in paraffin. Renal sections (4 *μ*m) were stained with PAS reagent (Solarbio, Beijing, China) to assess glomerular and tubular injury. Slices were analyzed with light microscopy, and images were captured with a digital camera.

Glomerulosclerosis was defined as the presence of a dense, abundant deposition of PAS staining positive at the glomerular tufts, with the occlusion of capillary loops and segmental hyalinization; damage in tubule-interstitial and arteriolar regions was defined as the presence of dilatation, protein cylinders, and atrophy [11]. FN expression in kidney tissue was detected by immunohistochemistry (IHC) of prepared sections. Briefly, after being deparaffinized and rehydrated, sections were treated with pH 9.0 Tris-EDTA (Zhongshan Golden Bridge Biotechnology, Beijing, China) for antigen retrieving with a microwave and were treated with 3% hydrogen peroxide (Zhongshan Golden Bridge Biotechnology, Beijing, China) for blocking endogenous peroxide. The sections were incubated with primary antibody (fibronectin, Proteintech, Chicago, IL, USA) at 4°C overnight. Rinsing with PBS, sections were added with a horseradish peroxidase- (HRP-) labeled secondary antibody (Zhongshan Golden Bridge Biotechnology, Beijing, China) at room temperature for 30 min. After the detection step with diaminoben (DAB) substrate (Zhongshan Golden Bridge Biotechnology, Beijing, China) and nuclei restaining with hematoxylin, slices were observed and captured.

### 2.11. Kidney Electron Microscopy Analysis

Renal cortices were fixed in 2% glutaraldehyde for 1 h. After three washes with PBS, the samples were postfixed with 1% osmium tetroxide in cacodylate buffer (pH 7.2) for 2 h. Subsequently, the samples were dehydrated in acetone and embedded in 812 epoxy resin (SPI supplies, West Chester, PA, USA). Ultrathin sections (60-80 nm) were cut, double-stained with uranyl acetate and Reynolds lead citrate, and examined with a transmission electron microscope (HITACHI, Tokyo, Japan).

### 2.12. Enzyme-Linked Immunosorbent Assay (ELISA) Measurements

The serum levels of IL-1*β*, IL-6, IL-10, TNF-*α*, TGF-*β*, and HGF were measured by ELISA. A portion of a 1.5 ml peripheral blood sample was collected via the venous route and centrifuged at 5000 r/min at 4°C for 10 min. The serum was collected and stored at -80°C for the test. The differences in the levels of IL-1*β*, IL-6, IL-10, TNF-*α*, TGF-*β*, and HGF (Beijing Qisong Biotech, Beijing, China) were simultaneously quantitatively measured strictly according to the manufacturer's instructions. The experiments were all performed in triplicate.

### 2.13. MSC Detection

The cryosections (5 *μ*m) were stained with DAPI (Solarbio, Beijing, China) and mounted. Slices were observed by fluorescence microscopy. Images were captured with a digital camera and virtually merged.

### 2.14. HK-2 Cell Culture and Coculture with MSCs

HK-2 cells were from Cell Bank, Shanghai Institutes for Biological Sciences (Chinese Academy of Sciences, Shanghai, China), cultured in epithelial cell medium (EpiCM, ScienCell, Cedro, CA, USA), and maintained at 37°C in an environment containing 5% CO_2_. Digestion was performed using 0.1% trypsin/0.1% EDTA. When monolayer cell confluence was observed, the cells were subjected to passage at a ratio of 1 : 3.

To generate a coculture system of HK-2 and MSCs, the medium for coculture was established so that both cell types could be cultured in the same medium conditions. Therefore, a 1 : 1 mixture of MCM and EpiCM was prepared.

**Figure 2:**
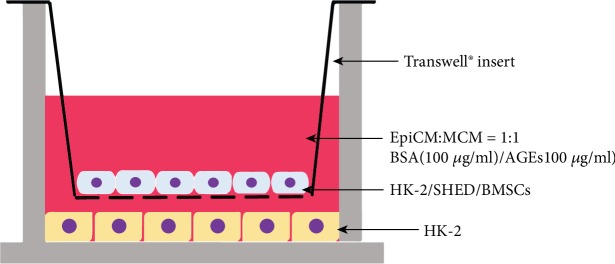
Coculture in transwell. HK-2 cells were first seeded in 6-well plates. The next day, suitable transwells were inserted, and MSCs or HK-2 (for control) cells were seeded on the transwell membrane.

HK-2 cells were first seeded in 6-well plates (5 × 10^5^/well) in EpiCM to adhere. The next day, HK-2 cells were cultured in coculture medium containing 100 *μ*g/ml BSA (Lablead Biotech, Beijing, China) or 100 *μ*g/ml AGEs (BioVision, San Francisco, CA, USA). Additionally, suitable transwells (0.4 *μ*m, Corning Incorporated, Corning, NY, USA) were inserted, and HK-2 cells, SHED, or BMSCs (1 × 10^5^/well) were seeded on the membranes of the transwells, as shown in Figure 2. The fresh cocultured medium was changed every day (see Figure 2). After 3 days of coculture, RNA and protein were extracted from HK-2 cells as described below.

### 2.15. HK-2 Cell RNA Expression

Total RNA was extracted from cocultured HK-2 cells and reverse transcribed into cDNA as previously indicated. cDNA was amplified by real-time PCR using the following primers (forward primer, reverse primer): GADPH—5′-GAAGGTGAAGGTCGGAGTC-3′, 5′-GAGATGGTGATGGGATTTC-3′; E-cadherin—5′-TACACTGCCCAGGAGCCAGA-3′, 5′-TGGACCAGTGTCCGGATTA-3′; *β*-cadherin—5′-GCTGAAGGTGCTATCTGTCTGCTC-3′, 5′-TGAACAAGACGTTGACCTTGGATCTG-3′; *α*-SMA—5′-CTGGCCGAGATCTCACTGACTA-3′, 5′-GCCCATCAGGCAACTCGTAA-3′; FSP-1—5′-CAGATAAGCAGGCCGAAAA-3′; and Fn—5′-TGCCTTGCACGATGATATGGA-3′, 5′-CTTGTGGGTGTGACCTGAGTGAA-3′.

### 2.16. Western Blot Analysis

Protein from cocultured HK-2 cells was extracted by RIPA (Solarbio, Beijing, China) according to the manufacturer's instructions, and the protein concentration was quantified using the Bradford method (Beyotime, Shanghai, China). Each protein lysate (30 *μ*g) was run on a 10% sodium dodecyl sulfate- (SDS-) polyacrylamide gel (Solarbio, Beijing, China) and transferred to a nitrocellulose membrane (Millipore, Shanghai, China). Membranes were blocked with 5% skim milk (Lablead Biotech, Beijing, China) in Tris-buffered saline (pH 7.4) containing 0.1% Tween 20 (TBST, Solarbio, Beijing, China) for 1 h at room temperature and then incubated for 16 h at 4°C with primary antibodies. Protein levels were normalized to *β*-actin levels. The primary antibodies used in this study were as follows: mouse polyclonal antibody to *β*-actin (1 : 1000; Proteintech, Chicago, NY, USA), rabbit polyclonal antibody to vimentin (1 : 1000; Abcam, Cambridge, UK), rabbit polyclonal antibody to FN (1 : 1000; Proteintech, Chicago, IL, USA), and mouse monoclonal antibody to E-cadherin (1 : 500; Proteintech, Chicago, USA). After extensive washes, the membranes were incubated with horseradish peroxidase-conjugated secondary antibody for 2 h at room temperature in 5% skim milk/TBST. ECL (Solarbio, Beijing, China) was used for detection.

### 2.17. Statistical Analysis

All data are presented as the mean ± standard deviation (SD). Statistical analysis was performed with SPSS software (version 17.0; SPSS Inc., Chicago, IL, USA). Student's *t*-test was used to compare the variables before and after administration. For multiple comparisons, one-way analysis of variance (ANOVA) was applied. Statistical significance was set at *P* < 0.05.

## 3. Results

### 3.1. MSCs Culture and Identification

**Figure 3:**
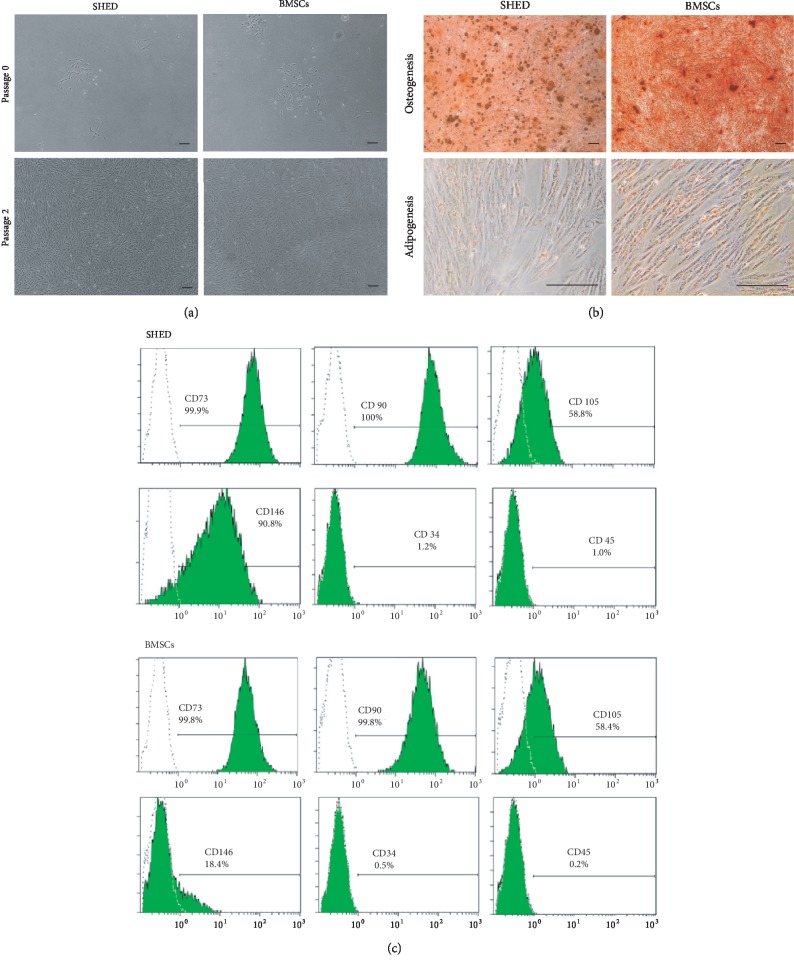
Characterization of SHED and BMSCs. (a) SHED and BMSCs showed fibroblast-like morphologies. (b) Multilineage differentiation potency including osteogenesis, as identified by Alizarin Red staining, and adipogenesis, as identified by Oil Red O staining. Bar = 100 *μ*m. (c) Flow cytometry of SHED and BMSCs. SHED expressed low levels of CD34 (1.2%) and CD45 (1.0%) and high levels of CD73 (99.9%), CD90 (100.0%), CD105 (58.8%), and CD146 (90.8%). BMSCs expressed low levels of CD34 (0.5%) and CD45 (0.2%) and high levels of CD73 (99.8%), CD90 (99.8%), CD105 (58.4%), and CD146 (18.4%).

SHED and BMSCs exhibited typical fibroblast-like morphologies. The identity of SHED or BMSCs was confirmed by differentiation into osteogenic or adipogenic cells (see Figure 3). In addition, BMSCs and SHED were identified by surface markers CD73(+), CD90(+), CD105(+), CD146(+), CD34(-), and CD45(-) by flow cytometry.

### 3.2. Effects of SHED on Physical and Biochemical Parameters

2 to 4 weeks after diabetes induction, the blood glucose levels of 36 of the 39 GK rats met the conditions for DN. Six rats were randomly chosen for cell tracking. In addition, 30 GK rats were randomly divided into three groups (SHED group: *n* = 12, PBS group: *n* = 8; and BMSCs group: *n* = 10).

After treatment administration, fasting blood glucose levels decreased significantly during the treatment period in both the SHED and BMSCs groups, while nonfasting blood glucose levels only decreased at two weeks after administration in the BMSCs group and decreased markedly at two weeks, three weeks, and seven weeks after administration in the SHED group (see Table 1).

In addition, compared to the normal group, GK rats presented increased serum cholesterol and serum triglyceride levels. At eight weeks after administration, serum triglycerides increased in the PBS group but remained stable in the BMSCs and SHED groups. In addition, SHED and BMSC treatment suppressed the 24 h urinary albumin level and kidney to bodyweight index after administration (see Table 2).

### 3.3. Effects of SHED on Renal Gene Expression

**Figure 4:**
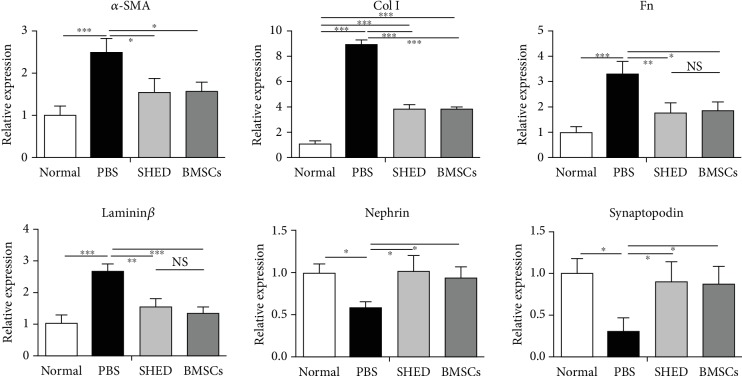
Effects of MSCs on *α*-SMA, Col I, FN, laminin *β*, nephrin, and synaptopodin mRNA expression in kidney tissue measured by real-time PCR. ^∗^*P* < 0.05, ^∗∗^*P* < 0.01, and ^∗∗∗^*P* < 0.001. The *α*-SMA, Col I, FN, and laminin *β* levels were obviously higher in PBS group than in the other three groups, and Col I level was lower in the SHED and BMSCs groups than in the PBS group. Nephrin and synaptopodin were lower in the PBS group than in the other three groups.

As shown in Figure 4, real-time PCR analysis showed that SHED administration significantly downregulated the diabetic-induced increases in *α*-SMA, collagen I, Fn, and laminin *β* expression in the renal cortex. In contrast, SHED administration significantly upregulated the diabetic-induced decreases in nephrin and synaptopodin expression in the renal cortex. Additionally, there was no difference in renal gene expression between the SHED group and BMSCs group (see Figure 4).

### 3.4. Effects of SHED on Renal Histological Changes

PAS staining showed similar changes in the PBS group, differing from the normal group in terms of the glomeruli and tubules, which displayed glomerular sclerosis, mesangial expansion, tubular dilatation, and protein cylinder by light microscopy. However, the extent of such changes in the glomeruli and tubules was improved in the SHED and BMSCs groups. Only a small amount of glomerulosclerosis and tubular dilatation was observed in the SHED group and BMSCs group. Further improvements in the structures or nearly normal structures of glomeruli and tubules were observed in the SHED and BMSCs groups. In addition, immunostaining for FN increased significantly in the PBS group and was remarkably reduced by SHED and BMSCs treatment.

**Figure 5:**
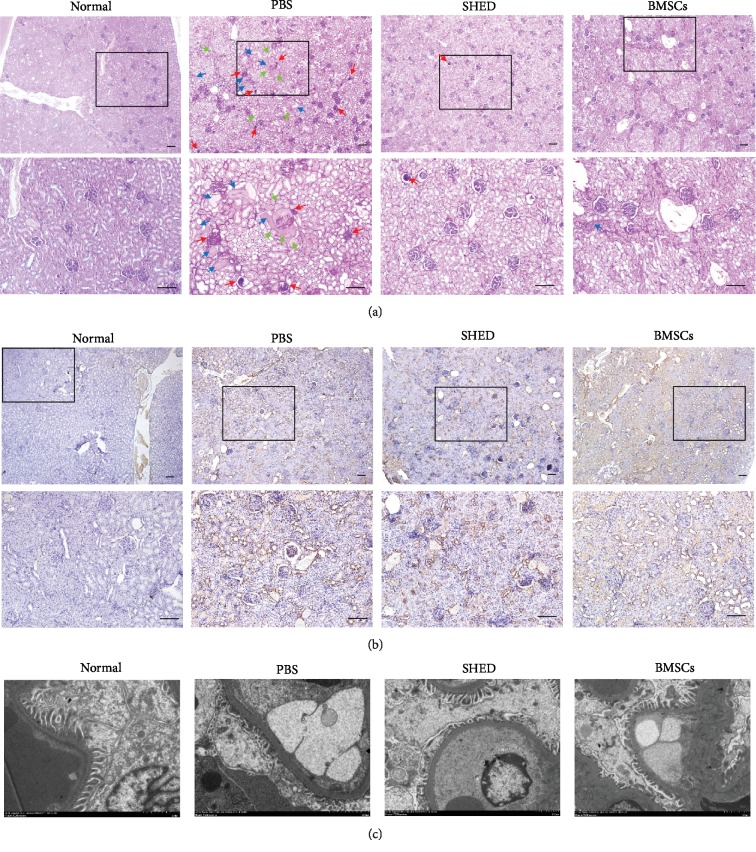
Effects of SHED on renal histopathological changes. (a) Representative images of PAS staining in the four groups. The PBS group displayed significant glomerular sclerosis, mesangial expansion, and tubular dilatation, while improvements in the glomeruli and tubules were observed in the SHED and BMSCs groups. (red arrow: glomerular sclerosis and mesangial expansion; blue arrow: tubular dilatation and protein cylinders; green arrow: renal tubular vacuolar degeneration). (b) IHC analysis of FN. FN increased significantly in the PBS group and remarkably decreased in the SHED and BMSCs groups. (c) Electron microscopy. The GEM was obviously thickened, and the foot processes of the podocytes were condensed and missing or in disarray in the PBS group. The thickness of the GEM and the foot processes of the podocytes improved in the SHED group and BMSCs group. Bar = 100 *μ*m.

Furthermore, GBM was obviously thickened, and the foot processes of the podocytes were condensed and missing or in disarray in the PBS group compared to the normal control. The thickness of GBM in both MSCs groups was between the thickness of the normal group and that of the PBS group. Additionally, the abnormalities of the foot process were improved in the SHED group and BMSCs group (see Figure 5).

### 3.5. Effects of SHED on Serum Cytokine Levels

**Figure 6:**
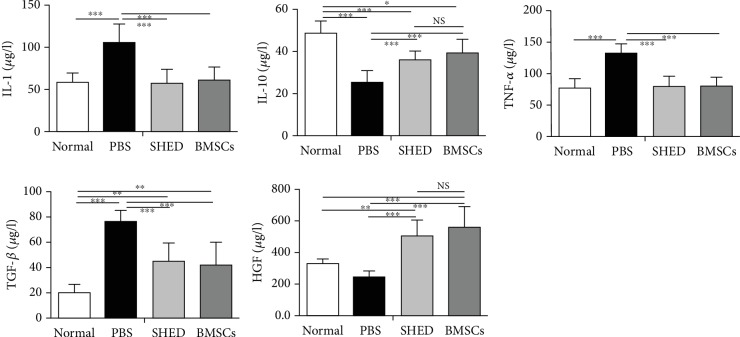
Serum IL-1, IL-10, TNF-*α*, TGF-*β*, and HGF levels were determined by ELISA. ^∗^*P* < 0.05, ^∗∗^*P* < 0.01, and ^∗∗∗^*P* < 0.001. IL-1 and TNF-*α* were clearly higher in the PBS group than in the other groups, and TGF-*β* level was lower in the SHED and BMSC groups than in PBS group; IL-10, TGF-*β*, and HGF levels in the SHED group and BMSC group were between those in the normal group and those in the PBS group.

In the PBS group, the levels of IL-1 and TNF-*α* increased significantly in comparison to those in the other three groups after cell transplantation. The levels of IL-10 and TGF-*β* in the BMSC and SHED groups were between those in the normal group and those in the PBS group. In the SHED and BMSC groups, the level of HGF considerably increased compared with that in the normal control group, while the level of HGF in the PBS group was the lowest of the four groups (see Figure 6).

### 3.6. SHED Tracking

**Figure 7:**
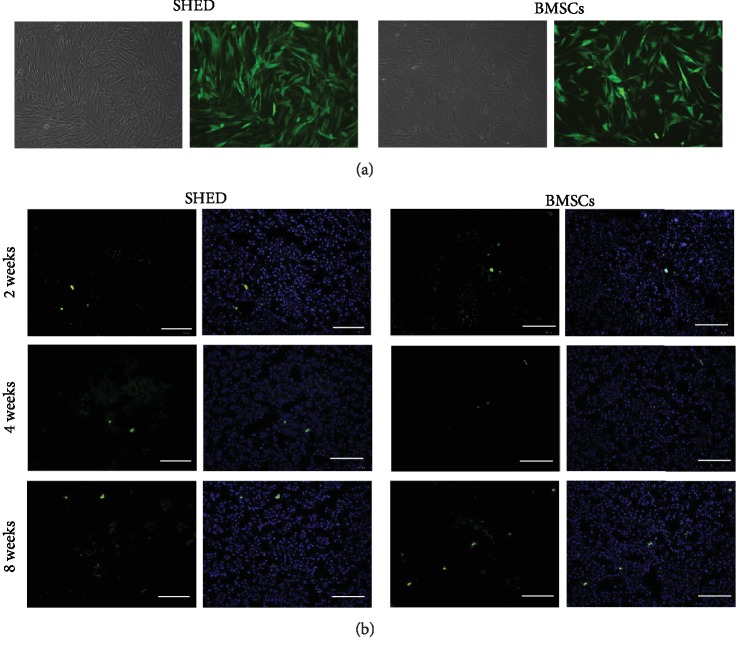
SHED engraftment in a diabetic kidney. (a) More than 80% of MSCs exhibited green fluorescence 3 days after transduction by lentiviral vector at an MOI of 100. (b) Representative micrograph of kidney tissue from diabetic rats. GFP-SHED/GFP-BMSCs (green) merged with DAPI (blue). Bar = 100 *μ*m.

Transduction of GFP into MSCs by lentiviral vector resulted in a high transduction efficiency, confirmed by nearly 80% of BMSCs and SHED exhibiting green fluorescence 3 days after transduction. At 2, 4, and 8 weeks after administration, GFP-BMSCs and GFP-SHED were found in the glomerular and tubulointerstitial regions (see Figure 7).

### 3.7. Effects of SHED on HK-2 Cultured with AGEs

Real-time PCR analysis showed that HK-2 cells cocultured with BMSCs or SHED significantly reversed the AGE-induced increases in *α*-SMA, Fn, and vimentin expression. In contrast, MSC administration significantly reversed the diabetes-induced decreases in E-cadherin and *β*-catenin expression.

**Figure 8:**
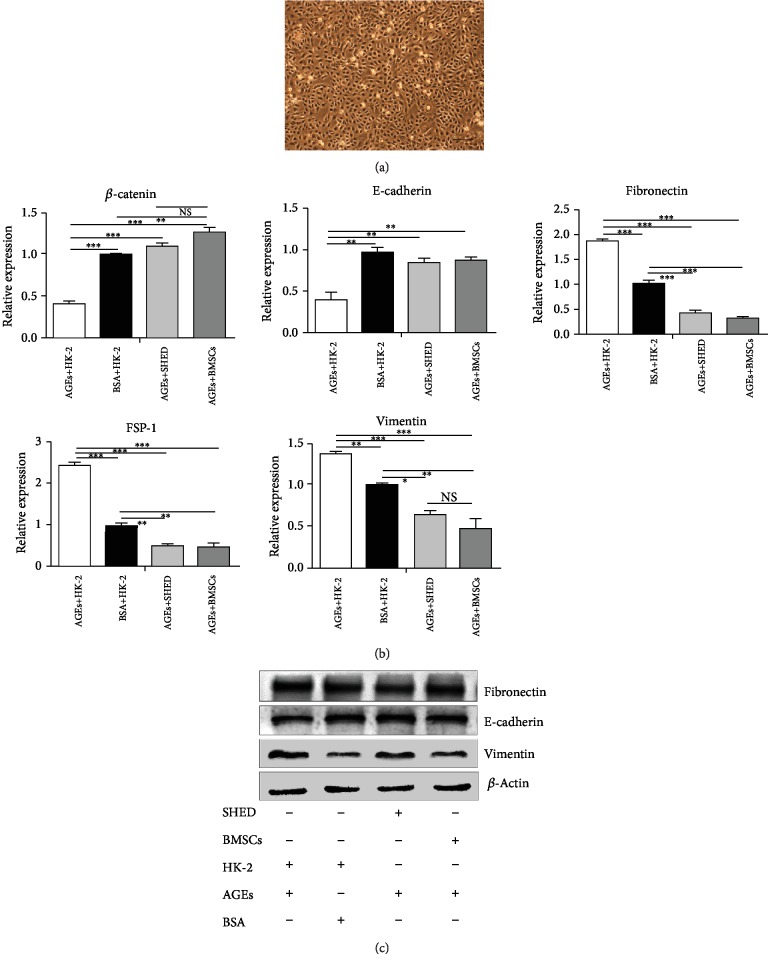
Effects of SHED on the EMT of HK-2 cells induced with AGEs. (a) HK-2 cells (P2) showed smooth-edged contours and a cobblestone-like morphology. (b) Effects of MSCs on *β*-catenin, E-cadherin, fibronectin, FSP-1, and vimentin mRNA expression by real-time PCR. ^∗^*P* < 0.05, ^∗∗^*P* < 0.01, and ^∗∗∗^*P* < 0.001. (c) Effects of MSCs on E-cadherin, fibronectin, and vimentin protein expression by western blot.

In addition, western blot analysis showed that HK-2 cells cocultured with BMSCs or SHED significantly reversed the AGE-induced increases in Fn and vimentin. In contrast, MSC administration significantly reversed the AGE-induced decreases in E-cadherin. Additionally, there were no differences in the HK-2 cells cultured with AGEs between the SHED and BMSC groups by real-time PCR or western blotting (see Figure 8).

## 4. Discussion

DN is one of the most serious complications of DM. However, there is no specific or efficacious drug treatment for DN. As a potential strategy, MSC therapy has received much attention over the years. Previous studies have indicated that BMSCs, ADSCs, and UCB-MSCs attenuate DN in STZ-induced animals [27–29].

In our study, the present data clearly showed that SHED administration ameliorated diabetic renal injury, including hyperglycemia, hyperlipidemia, increased proteinuria, a fractional mesangial area, ECM accumulation, and unbalanced activation of cytokines in GK rats and EMT in HK-2 cells. Additionally, BMSCs were used as the positive control, and there were no significant differences in the effects of SHED and BMSCs on DN.

The flow cytometry analysis in this study showed that SHED and BMSCs appeared to differ in their expression of CD146. CD146 (also known as melanoma cell adhesion molecule) is an antigen that is expressed on almost all kinds of epithelial cells, activated T cells, and dendritic cells [30, 31]. Recent reports showed that the expression of CD146 by MSCs had no significant effects on MSC characters [32]. Though some studies found CD146+ mesenchymal stem cells display greater therapeutic potential than CD146– for some other diseases, we found no significant differences in the effects of SHED and BMSCs on DN. And further study was needed.

Clinical studies have shown that the optimal number of MSCs administered for DM and its related complications is 3 × 10^6^/kg, which is not small [33–35]; hence, the potential for proliferation of the seed cell for MSC-based therapy is extremely important. Previously, the expansion rates and proliferation rates of SHED were found to be significantly higher than those of BMSCs. BMSCs need significantly more time to double, with a mean value of 125.24 ± 1.01 h, while the time required for the doubling of SHED is 44.68 ± 3.12 h [36]. Additionally, SHED are more accessible for clinical application by noninvasive retrieval and are discarded as medical waste. Fewer ethical concerns exist. Therefore, SHED are ideal seed cells for DM and related complications.

In our research, fasting blood glucose levels decreased significantly after 8 weeks of SHED and BMSC transplantation, while nonfasting blood glucose levels decreased significantly only at 2 weeks, 3 weeks, and 7 weeks after SHED administration, a result that differed from previous studies showing that nonfasting blood glucose levels decreased significantly during the entire treatment. One possible explanation is the experimental animal model used in the studies. The animal model used in this study is GK rats (T2DM model), while in previous studies, STZ-induced rats or mice were applied as T1DM or T2DM animal model. T1DM is characterized by defective *β*-cell secretory function while, T2DM, the most common type of DM, is characterized by insulin resistance and defective *β*-cell secretory function [37]. GK rats, a nonobese and spontaneous T2DM experimental model, have been widely used to investigate the progress and the treatment of T2DM and its complications, especially DN. GK rats were obtained via repetitive selection of breeding of glucose-intolerant Wistar rats exhibiting peripheral insulin resistance and defective insulin secretory function [38, 39]. However, the mechanism of STZ-induced DM is impairment of islet targeting. Another explanation for the discrepant results may be nonnegligible differences between individual GK rats, so future studies with large sample sizes are necessary. Moreover, the delivery route of MSCs [27], the number of cells [29, 40], and the dose of infusion may also contribute to the result of MSC administration. The beneficial effect of a single infusion of MSCs in ameliorating hyperglycemia in diabetic rats was maintained only for a period not exceeding 4 weeks [41].

Dyslipidemia has been reported to be highly prevalent among subjects with nephropathy [42].

Moreover, dyslipidemia accelerates the rate of renal damage. The mechanism behind this action has not been fully elucidated, but the notion that lipids may damage the vascular, mesangial, and tubular cells of the kidney has been proposed [43]. We found that SHED downregulated serum triglycerides instead of serum cholesterol, while the effects of SHED on the low high-density lipoprotein cholesterol (HDL-C) concentration and small, dense, low-density lipoprotein (sdLDL) particles need further study.

Although the effects of SHED on hyperglycemia and hyperlipidemia remain to be further explored, the renoprotective effects of SHED were achieved. We observed that SHED transplantation at 8 weeks after administration attenuated the progression of diabetic renal injury as evidenced by biochemical, molecular biological, and histopathological results and electron microscopy. Our results are consistent with those of previous studies highlighting the renal protective effects of BMSCs extracted from mice, rats, or even humans. ADMSCs from rats and USB-SCs from humans inhibit albuminuria and kidney damage in STZ-induced diabetic rats and mice [44–46].

Inflammatory factors promote the accumulation and activation of macrophage and accelerate the progression of diabetic renal injury. IL-1, IL-6, and tumor necrosis factor-*α* (TNF-*α*) not only alter the expression of chemotactic factors and adhesion molecules but also alter intraglomerular hemodynamics mediated by prostaglandins, leading to increased vascular endothelial cell permeability and hyaluronan production by renal tubular epithelial cells and leading to promoted ECM accumulation and mesangial expansion. In contrast, IL-10, the main anti-inflammatory and immunosuppressive cytokine, produced by several types of immune cells, plays a key role in the regulation of immune responses, inhibiting leukocyte infiltration and tissue damage in DN [44]. T2DM is with respective activation of much inflammatory cytokines. We supposed that SHED administration via the tail vein downregulated the serum levels of IL-1 and TNF-*α* and upregulated those of IL-10 in DN rats, not only improving insulin resistance but also affecting the renal immune response through inflammatory cytokines. In addition, in this study, SHED downregulated the serum levels of TGF-*β* while increasing the serum levels of HGF, thereby preventing glomerulosclerosis and tubule-interstitial injury in DN. TGF-*β*1 has been reported to contribute to cell hypertrophy and the increased synthesis of collagen, ultimately leading to glomerulosclerosis and tubule-interstitial injury during DN development, while HGF has been reported to ameliorate DN by blocking the profibrotic actions of TGF-*β*1 [45].

AGEs (crosslinks with collagen I that contributes to microvascular complications) are a novel risk factor in the pathogenesis of end-stage renal disease resulting from DN [46]. Sustained AGE exposure has been reported to typically cause renal tubular epithelia cells to undergo EMT, losing their epithelial phenotypes and acquiring mesenchymal, fibroblast-like properties [47]. In addition, hUSB-MSC-conditioned media restrain TGF-*β*1-activated EMT in NRK-5RE cells, reversing the high mRNA expression of collagen I and Hsp47 and reversing the low mRNA expression of E-cadherin and BMP-7 [28]. Therefore, we explored the effects of SHED on AGE-activated EMT in HK-2 cells. In this study, a coculture system was constructed with 0.4 *μ*m transwell so that cells cultured in the upper chambers could not pass through the polycarbonate membrane into the lower chambers. Meanwhile, this system enabled cells from different chambers to freely exchange substances. PCR and western blotting showed that SHED significantly attenuated the AGE-induced EMT of HK-2 cells.

We suspect that the effects of SHED on AGE-induced EMT may be related to SHED engraftment in the kidney, not only regulating the local inflammatory environment but also attenuating the EMT in the renal tubular epithelium. In the present study, the GFP data confirmed the engraftment of a few tail vein-injected SHED into the glomeruli and tubulointerstitial tissue at 2 weeks, 4 weeks, and 8 weeks after administration which agrees with the former researches [28, 29]. Although the exact mechanism involved in stem cell homing to injury remains elusive, hypoxia, inflammation, and high glucose, all of which are present in diabetic kidneys, may induce the migration and proliferation of MSCs. Although embryonic stem cells can differentiate into tubular cells or mesonephric ducts, it is widely accepted that the paracrine mechanism of MSCs plays an essential role in their protective effects [48].

## 5. Conclusion

In conclusion, SHED prevent kidney injuries in DN including hyperglycemia, hyperlipidemia, increased proteinuria, ECM accumulation, and fractional mesangial area as well as type II EMT possibly through a paracrine action and suggest that SHED may become an effective therapeutic modality for ameliorating DN, the leading cause of end-stage renal disease.

## Figures and Tables

**Table 1:** Nonfasting blood glucose levels and fasting blood glucose levels in the 4 groups of rats. Values are expressed as the mean ± SD. ^∗^*P* < 0.05 vs. the normal group. ^#^*P* < 0.05 vs. 0 week in the same group. Fasting blood glucose levels decreased significantly after treatment administration in both the SHED and BMSCs groups, while nonfasting blood glucose levels only decreased at two weeks after administration in the BMSCs group and decreased markedly at two weeks, three weeks, and seven weeks after administration in the SHED group.

Week	Nonfasting blood glucose (mM)				Fasting blood glucose (mM)			
	Normal	PBS	SHED	BMSCs	Normal	PBS	SHED	BMSCs
0	5.83 ± 0.25	14.93 ± 2.8^∗^	15.13 ± 3.81^∗^	14.41 ± 2.41^∗^	4.68 ± 0.39	13.93 ± 5.80^∗^	13.89 ± 3.66^∗^	15.78 ± 4.14^∗^
1	6.20 ± 0.42	13.52 ± 5.32^∗^	11.13 ± 3.79^∗^	12.30 ± 4.37^∗^	5.23 ± 0.42	10.43 ± 4.11^∗^	8.43 ± 1.72^∗^^#^	8.66 ± 1.85^∗^^#^
2	6.04 ± 0.27	13.61 ± 5.31^∗^	10.86 ± 3.69^∗#^	11.13 ± 3.66^∗#^	5.04 ± 0.28	9.67 ± 4.52^∗^	8.46 ± 1.35^∗#^	9.24 ± 2.30^∗#^
3	6.06 ± 0.49	13.01 ± 4.69^∗^	11.03 ± 3.30^∗#^	14.14 ± 5.89^∗^	5.39 ± 0.49	11.66 ± 4.83^∗^	8.28 ± 2.24^∗#^	9.61 ± 4.49^∗#^
4	5.92 ± 0.50	13.37 ± 5.29^∗^	11.94 ± 5.02^∗^	12.78 ± 4.26^∗^	4.92 ± 0.51	11.34 ± 6.52^∗^	7.79 ± 1.92^∗#^	8.09 ± 1.41^∗#^
5	5.84 ± 0.30	18.91 ± 9.04^∗^	13.02 ± 5.77^∗^	15.26 ± 6.96^∗^	5.14 ± 0.46	13.92 ± 5.69^∗^	11.06 ± 4.89^∗^	9.31 ± 4.11^∗#^
6	5.88 ± 0.83	14.30 ± 3.50^∗^	12.03 ± 4.17^∗^	12.23 ± 4.09^∗^	5.88 ± 0.54	11.73 ± 5.36^∗^	9.74 ± 3.87^∗#^	9.99 ± 3.19^∗#^
7	6.04 ± 0.85	13.61 ± 5.31^∗^	11.36 ± 3.18^∗#^	11.96 ± 3.62^∗^	5.50 ± 0.27	10.74 ± 4.97^∗^	10.38 ± 3.45^∗#^	10.10 ± 2.96^∗#^
8	6.16 ± 0.46	12.70 ± 4.66^∗^	12.71 ± 6.22^∗^	14.10 ± 4.56^∗^	5.56 ± 0.66	13.44 ± 5.57^∗^	9.96 ± 4.57^∗#^	11.46 ± 3.53^∗#^

**Table 2:** The physical and biochemical parameters of the four groups of rats before and after administration. Values are expressed as the mean ± SD. ^∗^*P* < 0.05 vs. the normal group, ^∗∗^*P* < 0.05 vs. both the normal and PBS groups, and ^#^*P* < 0.05 vs. before in the same group.

	Normal		PBS		SHED		BMSCs	
	Before	After	Before	After	Before	After	Before	After
Body weight (g)	492.52 ± 22.98	541.84 ± 18.47	446.67 ± 49.22^∗^	475.22 ± 59.64^∗^	448.67 ± 23.47^∗^	486.63 ± 26.00^∗^	456.57 ± 40.13^∗^	505.11 ± 46.51^∗^
Serum cholesterol (mM)	1.69 ± 0.23	1.56 ± 0.35	2.09 ± 0.11^∗^	3.45 ± 0.78^∗#^	1.94 ± 0.44^∗^	3.66 ± 0.69^∗#^	2.04 ± 0.16^∗^	3.45 ± 0.78^∗#^
Serum triglycerides (mM)	1.34 ± 0.21	1.51 ± 0.25	1.61 ± 0.76	2.74 ± 1.07^∗^	1.04 ± 0.73	1.68 ± 0.80	1.31 ± 1.01	1.04 ± 0.73
Urinary albumin (mg/24 h)	0.13 ± 0.06	0.12 ± 0.07	1.76 ± 0.78^∗^	4.00 ± 0.80^∗#^	1.64 ± 0.88^∗^	1.38±0.31^∗∗^	1.94 ± 0.52^∗^	1.58±0.23^∗∗^
Kidney/body weight% (mg/g)		6.07 ± 1.00		9.62 ± 1.50^∗^		7.96±1.30^∗∗^		8.28±1.06^∗∗^

## Data Availability

The table and figure data used to support the findings of this study are included within the article.

## Abbreviations

MSCs: Mesenchymal stem cells
DN: Diabetic nephropathy
SHED: Stem cells from human exfoliated deciduous teeth
GK rats: Goto-Kakizaki rats
IHC: Immunohistochemistry
GFP: Green fluorescence protein
AGEs: Advanced glycation end products
EMT: Epithelial-mesenchymal transition
HK-2 cells: Human proximal tubular epithelial cells
DM: Diabetes mellitus
T1DM: Type 1 diabetes mellitus
T2DM: Type 2 diabetes mellitus
ESRD: End-stage renal disease
UAE: Urinary albumin excretion
GEM: Glomerular basement membrane
STZ: Streptozotocin
RAGE: Receptor of advanced glycation end products
RAAS: Renin-angiotensin-aldosterone system
BMSCs: Bone marrow-derived mesenchymal stem cells
PBS: Phosphate-buffered saline
*α*-MEM: *α*-Modified Eagle's medium
FBS: Fetal bovine serum
MCM: Mesenchymal cell-conditioned medium
MOI: Multiplicity of infection
GAPDH: Glyceraldehyde 3-phosphate dehydrogenase
Col I: Collagen I
PAS stain: Periodic acid-Schiff stain
FN: Fibronectin
ELISA: Enzyme-linked immunosorbent assay
IL-1: Interleukin-1
IL-10: Interleukin-10
TNF-*α*: Tumor necrosis factor-*α*
TGF-*β*: Transforming growth factor-*β*
HGF: Hepatocyte growth factor
EpiCM: Epithelial cell medium
SDS: Sodium dodecyl sulfate
HDL-C: High-density lipoprotein cholesterol
sdLDL: Small, dense, low-density lipoprotein
ADMSCs: Adipose-derived mesenchymal stem cells
USB-SCs: Umbilical cord blood-derived mesenchymal stem cells.
